# Accuracy and Precision of a Surgical Navigation System: Effect of Camera and Patient Tracker Position and Number of Active Markers

**DOI:** 10.2174/1874325001711010493

**Published:** 2017-05-31

**Authors:** Kenneth R. Gundle, Jedediah K. White, Ernest U. Conrad, Randal P. Ching

**Affiliations:** 1 Oregon Health & Science University, Department of Orthopaedics & Rehabilitation, Portland, USA; 2 Portland VA Medical Center, Operative Care Division, Portland, USA; 3 University of Washington Medical Center, Department of Orthopaedics & Sports Medicine, Washington, USA; 4 Seattle Children’s Hospital, Department of Orthopaedic Surgery, Washington, USA; 5 University of Washington Applied Biomechanics Laboratory, Washington, USA

**Keywords:** Surgical Navigation, Computer-assisted surgery, Bone tumors, Registration, Accuracy, Error

## Abstract

**Introduction:**

Surgical navigation systems are increasingly used to aid resection and reconstruction of osseous malignancies. In the process of implementing image-based surgical navigation systems, there are numerous opportunities for error that may impact surgical outcome. This study aimed to examine modifiable sources of error in an idealized scenario, when using a bidirectional infrared surgical navigation system.

**Materials and Methods:**

Accuracy and precision were assessed using a computerized-numerical-controlled (CNC) machined grid with known distances between indentations while varying: 1) the distance from the grid to the navigation camera (range 150 to 247cm), 2) the distance from the grid to the patient tracker device (range 20 to 40cm), and 3) whether the minimum or maximum number of bidirectional infrared markers were actively functioning. For each scenario, distances between grid points were measured at 10-mm increments between 10 and 120mm, with twelve measurements made at each distance. The accuracy outcome was the root mean square (RMS) error between the navigation system distance and the actual grid distance. To assess precision, four indentations were recorded six times for each scenario while also varying the angle of the navigation system pointer. The outcome for precision testing was the standard deviation of the distance between each measured point to the mean three-dimensional coordinate of the six points for each cluster.

**Results:**

Univariate and multiple linear regression revealed that as the distance from the navigation camera to the grid increased, the RMS error increased (p<0.001). The RMS error also increased when not all infrared markers were actively tracking (p=0.03), and as the measured distance increased (p<0.001). In a multivariate model, these factors accounted for 58% of the overall variance in the RMS error. Standard deviations in repeated measures also increased when not all infrared markers were active (p<0.001), and as the distance between navigation camera and physical space increased (p=0.005). Location of the patient tracker did not affect accuracy (0.36) or precision (p=0.97)

**Conclusion:**

In our model laboratory test environment, the infrared bidirectional navigation system was more accurate and precise when the distance from the navigation camera to the physical (working) space was minimized and all bidirectional markers were active. These findings may require alterations in operating room setup and software changes to improve the performance of this system.

## INTRODUCTION

Computer-assisted navigation technologies are being applied to an increasing range of applications with a unifying goal of improving surgical accuracy and precision. In orthopaedics, there have been significant efforts to utilize navigation as a means to potentially improve the safety of placing spinal pedicle and pelvic screws [1-4], for the precise placement of implants for joint replacements [5, 6], and for accurate resection of osseous tumors and subsequent reconstruction [6-10]. However, there are many opportunities for error to occur in these complex navigation systems [11].

Conceptually, the purpose of surgical navigation in oncology is to allow the surgeon to check accurate and precise locations in 3D space based on advanced imaging modalities. To achieve this goal, the images on a computed tomography (CT) or magnetic resonance imaging (MRI) scan must be linked in some fashion to the patient’s body in the operating room, and must be able to account for any motion during the procedure. The required components of a surgical navigation system are: a patient tracker (also known as a dynamic reference), which is attached for orthopaedic oncology purposes to the bone of interest; a camera that can record and interpret the location of the patient tracker and other tools along with associated navigation software.

A requisite feature of image-based surgical navigations systems is to link the physical space, which clinically is the patient and area of operative interest, with an image space such as a preoperative CT or MRI scan [12]. The linking process is known as registration. Image-to-patient registration process is the most critical step in surgical navigation^6^ and one of the greatest potential sources of error [13]. A common means of merging physical space to image space is paired-point (or point-to-point) registration: identifying and selecting the same reference points in both spaces with the navigation system then computing the 3D coordinate transformation between both spaces. The navigation system then computes the mean residual error (MRE) after this process, with values less than 1-2mm considered a successful registration [14]. The MRE, also known as the fiducial registration error (FRE), is a measure of overall goodness of fit in how the physical space and image space are merged. However, it is unclear what the MRE actually represents functionally, and it is not equivalent to the positional accuracy of the navigation system at targeted locations [15]. While an adequate MRE may be indicative of a reasonably good registration, it is not a sufficient measure of computer navigation accuracy [6, 11].

Numerous studies have reported the ability of surgical navigation systems to improve screw placement, or achieve a higher rate of placing components in a desired alignment [16, 17]. Such clinical validation is different from a controlled laboratory assessment of accuracy and precision of individual components and processes [11]. Additionally, there is a paucity of guidance in the literature on modifiable factors in the setup of the operating environment and surgical tactic. Hence, the purpose of this study was to investigate the impact of several modifiable variables on the accuracy and precision in an imaged-based bidirectional infrared navigation system.

## METHODS

A Stryker Navigation System II mobile tower (Stryker Corp., Kalamazoo, Michigan) was obtained and calibrated in situ prior to testing. The experimental setup is shown in Fig. (**1**). It consisted of the cameras, a patient tracker (dynamic reference base), and computerized-numerical-controlled (CNC) machined grid.

An accurate CNC-machined grid with known grid-point locations was used as our test reference tool. The grid has a series of indentations, that matches and accepts the navigation pointer tip, located at 10 mm increments. Precise measurements between the grid points were measured and confirmed with a MicroScribe MX 3D digitizing system (Solution Technologies, Inc., Oella, MD). The grid was securely mounted at a known distance from the patient tracker and camera. To establish the imaging space for the test, DICOM information was created based on a high-resolution scan of the grid surface (Epson Perfection 1650, Long Beach, CA), and imported into the OrthoMap 3D navigation software Version 1.0 (Stryker Corp., Kalamazoo, Michigan). The distances between grid points on the scan were verified *via* digital imaging analysis software (NIH Image-J, Bethesda, MD).

Three variables were studied: the distance from the camera to the patient tracker, the distance from patient tracker to the center of the physical space, and the number of active infrared markers in use on the patient tracker and pointer.

To test the effect of the distance from the camera to the patient tracker, three positions were selected: 150cm, 200cm, and 247cm (maximum allowable system distance). Given intraoperative space and line of site constraints, 150cm was the minimum practical distance for use. Beyond 247cm, the patient tracker and pointer were not visible to the camera and the system would not allow for tool registration, so this distance was selected as the upper limit.

To test the effect of the distance from patient tracker to the center of physical space, three positions were selected: 20cm, 30cm, and 40cm. In pilot testing, placing the patient tracker closer than 20cm from the center of physical space often led to the pointer making contact with the patient tracker during testing. The greatest relevant distance in the human body is femoral length, which averages 48cm in an adult male. For distal femur excisions and reconstructions in orthopaedic oncology, the patient tracker is placed in the greater trochanter. Given that a patient tracker is placed in the bone being operated on, 40cm is at the extreme of likely practical application, so it was selected as the upper limit.

To test the effect of number of active infrared markers, two scenarios were chosen: all markers active (11 total: 5 on patient tracker, 6 on pointer), and minimum markers active (7 total: 4 on patient tracker, 3 on pointer). The minimum number of markers required for the system to function was determined as follows: each infrared marker was iteratively covered with electrical tape. The navigation instrument assessment tool from the system menu was used to confirm that markers were not active once covered. For the patient tracker, if more than one marker was covered, the system would not recognize the tracker at all. The tracker also could not be recognized if the central, out of plane marker was covered. Therefore, the inferior marker was selected for the ‘minimum marker’ test. For the pointer, three markers was the maximum number that could be covered before the system would not recognize the pointer at all.

With three distances for camera to patient tracker, three distances from patient tracker to center of physical space, and two marker configurations, a total of 18 trials were completed. For each trial, the patient tracker and pointer were activated and calibrated according to manufacturer instructions. Next, point-to-point registration was done using the same four points for each trial, located at indentations in the corners of the CNC grid. The precise location of these four points was determined in the planning phase of the OrthoMap software, and the distances between them confirmed. The error output for registration error overall and at each point was recorded. No surface mapping was done.

Using the annotation point feature of the OrthoMap software, the pointer tool was placed into each indentation on the grid and selected, yielding 60 annotation points per trial (yielding 1,080 measured points over 18 trials). Then, to enable precision analysis at each of the four registration points, five additional annotation points were collected while maximally varying the angular orientation of the pointer with the pointer tip bottomed in the indentation. This process was repeated for all 18 trials, by one author (KRG), yielding three-dimensional (x, y, z) coordinates of all annotation points.

## STATISTICAL ANALYSIS

The indentation points on the grid allowed for distances from 10mm up to 140mm to be computed and compared against the known machined distances (also verified using a MicroScribe 3D digitizing system). Utilizing all rows, twelve measurements were selected for each 10mm increment between 10mm and 120mm, for a total of 144 measurements in each trial. Length measurement error (LME), the absolute difference between a measured length and the true length as confirmed by the MicroScribe, was calculated. Subsequently, the root mean square (RMS) error was calculated for each distance from 10mm to 120mm, for each of 18 trials. The primary outcome variable for accuracy was RMS error, which is a unitless measure.

Precision at the registration points was assessed using the five repeated measurements at each registration point. For each, the average three-dimensional coordinates were calculated, and the mean distance from this average as well as the standard deviation was computed. The dataset, therefore, consisted of mean distance and standard deviation for the four registration points, in each of the 18 trials. The primary outcome variable for precision was the standard deviation of the repeat measures.

Preplanned statistical analyses included univariate analysis of variance (ANOVA) assessing covariants of camera distance, distance from patient tracker to the grid, and whether all or minimal bidirectional infrared markers were active. A dummy variable was used for infrared marker status. A two-tailed Students’ t-test was used to compare the means with two groups.-Multivariate analysis by linear regression included all covariants with a p<0.15 on univariate analysis. Analysis was done in R version 3.2.2 (Vienna, Austria), and p-value of 0.05 chosen for significance.

## RESULTS

### Accuracy

**Fig. (2):**
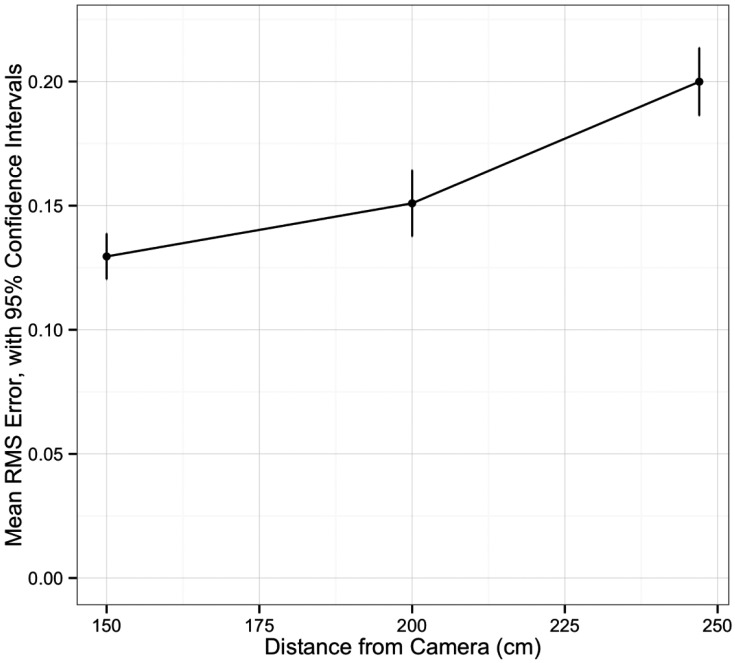
Average root mean squared error in navigation system, by distance from navigation camera.

**Fig. (3):**
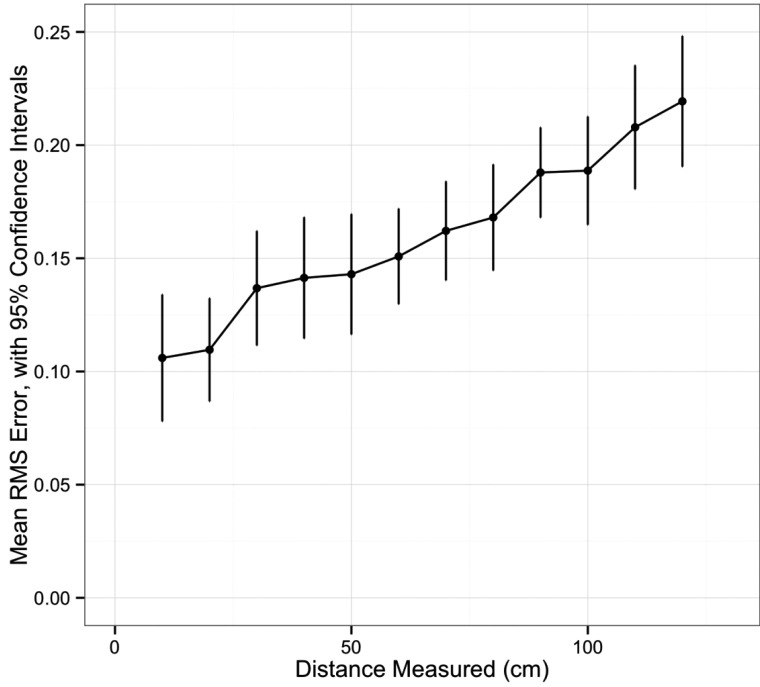
Average root mean squared error in navigation system, by distance measured.

Univariate analysis of navigation system accuracy is summarized in Table **1**. There was an increase in RMS error with increasing distance between the navigation system camera and the grid (p<0.001, (Fig. **2**). Having all markers active was associated with less error (p=0.03). The distance between the patient tracker and the grid did not affect accuracy (p=0.36). There was a difference in RMS error among the distances ranging from 10 to 120mm, with a trend for increased error at longer distances (p<0.001, (Fig. **3**).

For the multivariate linear regression, tracker distance was excluded based on univariate results. The final model included the distance from the camera (p<0.001), the distance being measured (p<0.001), and whether the maximum or minimum number of markers were active (p=0.001) as covariates (overall model: R^2^=0.58, p<0.001, (Table **2**).

### Precision

Univariate analysis of precision is summarized in Table **3**. Distance from camera to physical space (p=0.005) and whether the maximum or minimum number of markers were active (p<0.001) showed significant differences in precision. Patient tracker distance to physical space were similar in the three groups (p=0.97). Multivariate linear regression eliminated patient tracker as a significant covariate, with similar results to the univariate analysis. The final model is shown in Table **4** (overall model R^2^=0.53, p<0.001).

## DISCUSSION

This study investigated the accuracy and precision of an infrared bidirectional surgical navigation system in a controlled laboratory environment with paired-point registration. The variables studied were chosen for clinical relevance, as they may be affected by the operating room setup and intraoperative utilization: the distance from the navigation system camera and patient tracker device to the physical space (in this case, a precision CNC-machined grid),-and whether all or a minimum number of infrared markers on the navigation pointer and patient tracker were active. The final multivariate analyses found that these variables accounted for 58% of the variance in accuracy and 53% of the precision in the surgical navigation system.

Both accuracy and precision were negatively affected at increased distances between the navigation system camera and the grid. Beyond 247cm this system would not allow for the registration of the patient tracker or pointer, so that served as the upper limit. However, the finding that accuracy and precision were improved at the 150cm distance has significant implications. Should these findings be confirmed, an appropriate apparatus (such as a ceiling-mount) to facilitate the camera to be closer to the operating environment would be advisable.

The distance from the patient tracker device to the working space did not affect accuracy or precision between 20 and 40cm from the grid. For the vast majority of clinical applications, this suggests that the patient tracker may be placed at a point of convenience within the osseous structure of interest without concern for an impact on this navigation system’s performance. In contrast, the length of the distance measured adversely affected accuracy. As the measured distance rose from 10 to 120mm, the mean RMS error increased significantly. This may have implications for orthopaedic oncology applications, such as measuring resection lengths, though this study was not designed to assess the potential magnitude of this impact.

When the minimum number of markers was active we found significantly worse accuracy and precision compared to having all markers functioning. In the clinical use of navigation systems, it is common to hear an error tone signaling that the camera is visualizing insufficient markers on the pointer and or the patient tracker. In this event, the line of site may be blocked by the surgeon, assistant, or patient anatomy. The current iteration of software also does not actively display the number of visualized (active) markers for this navigation system. We chose to investigate the worse-case scenario of having the minimum required number of markers for the system to function; it is possible that having only one or two markers blocked would not significantly affect system performance. Adding the ability to visualize the number of working markers in real time, or increasing the total number of markers, may aid the surgeon in maximizing precision and accuracy.

As a lab-based model, a limitation of this study is its lack of direct clinical correlation. Whether the amount of error observed would impact clinical outcomes is unknown, and may be dependent on the surgical application. If the premise of surgical navigation is to improve outcomes through increased accuracy and precision, however, understanding and optimizing the variables the impact these parameters is an important aim. A strength of this study is that we examined variables largely within the control of the operating surgeon, or else feasible by changes in the navigation system software.

Another limitation is that only paired-point registration was utilized. This method of registration requires the surgeon to accurately touch points in the operative field that have been chosen on the preoperative imaging. Yau and associates studied repeated paired point registration by a single surgeon of lower extremity osseous landmarks with all soft tissues removed [18]. The average dispersion from anatomic landmarks ranged from 0.9 to 2.8 mm, with a projected maximal malalignment of only 1.3˚ in the coronal plane but up to 8.2˚ in the transepicondylar axis [18]. A follow-up study with two surgeons repeating registration and comparing selected anatomic sites to CT in a more clinically-applicable scenario with some soft-tissues intact showed an error of up to 24mm in selected osseous landmarks with significant intra-and inter-observer variability, though the authors suggest the impact on mechanical alignment is unlikely to be of clinical significance [19].

The navigation pointer fits concentrically into the indentation of our machined grid. Therefore these results approximate a best-case scenario for paired-point registration. A similar result may be obtained by inserting fiducials into patients preoperatively, as has been reported for neurosurgical and orthopaedic oncology applications, though this requires an additional procedure [20]. Surface mapping was not used in the current study, but as a supplement to paired-point registration it has shown reduced mean registration error [21]. Surface mapping requires the surgeon to select a cloud of points on bone, which the software then attempts to find a best fit for registration. This is generally an additional step, done after paired-point registration. A promising alterative to these registration schemes involves intraoperative imaging by fluoroscopy or CT to automate registration, though these systems can involve significant radiation exposure to the patient and operating room personnel, and require additional costly capital equipment [22]. The effect of distance between camera and surgical field, or the impact of minimum verse maximum marker visibility, has not to our knowledge been evaluated in these alternative systems.

Computer-assisted surgical navigation has shown potential in the resection and reconstruction of osseous tumors. Minimizing errors in image-to-patient registration is critical to the success of these systems. The results of this study suggest minimizing the distance from a surgical navigation system’s camera to the physical working space to improve its accuracy and precision. Also, in order to minimize potential errors, the navigation system should display in real time whether all infrared markers on the patient tracker and pointer are active. While limited by direct evidence of clinical relevance, this study provides recommendations for operating room setup and software features to improve the accuracy and precision of surgical navigation.

## Figures and Tables

**Fig. (1):**
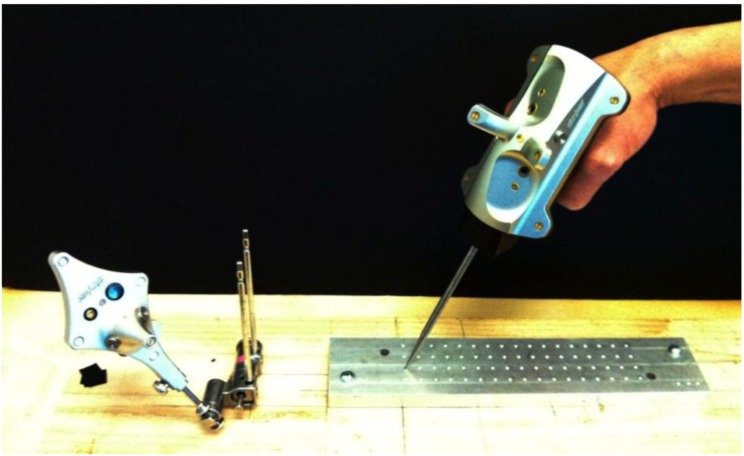
Experimental Setup, with patient tracker, pointer and machined grid.

**Table 1:** Summary of univariate analyses for accuracy data.

**Covariate**	**RMS error**	**95% CI**	**p value**
*Distance from Camera (cm)*			
150	0.13	0.12 - 0.14	
200	0.15	0.14- 0.16	
247	0.20	0.19 - 0.21	
			p<0.001
*Marker status*			
All active	0.15	0.14 - 0.16	
Minimum active	0.17	0.16 - 0.18	
			p=0.03
*Patient Tracker distance (cm)*			
20	0.17	0.16-0.18	
30	0.17	0.16-0.18	
40	0.14	0.13-0.15	
			p = 0.36

*t-test used to compare two groups. ANOVA used for more than two groups.

**Table 2:** Final model of multivariate analysis for accuracy data.

**Covariate**	**p value**	**Beta**	
	<0.001	0.57	
Distance from Camera	<0.001	0.48	
Marker Status	0.001	0.15	
			R^2^ = 0.58
			p<0.001

**Table 3:** Summary of Univariate Analyses for Precision Data.

**Covariate**	**SD**	**95% CI**	**p value**
*Distance from Camera*			
150	0.21	0.16 - 0.27	
200	0.36	0.27 - 0.44	
247	0.34	0.28 - 0.41	<0.01
*Marker status*			
All active	0.19	0.15 - 0.23	
Minimum active	0.42	0.37 - 0.47	
			<0.01
*Patient Tracker distance*			
20	0.31	0.22 - 0.40	
30	0.30	0.23 - 0.36	
40	0.31	0.24 - 0.38	
			0.97

*t-test used to compare two groups. ANOVA used for more than two groups.

**Table 4:** Final Model of Multivariate Analysis for Precision Data.

**Covariate**	**p value**	**Beta**	
Distance from Camera	<0.01	0.31	
Marker Status	<0.01	0.67	
			R^2^ = 0.53
			p<0.01

## LIST OF ABBREVIATION

ANOVA: = Analysis of variance
CNC: = Computerized-numerical-controlled
CT: = Computed tomography
FRE: = Fiducial registration error
MRE: = Mean residual error
MRI: = Magnetic resonance imaging
RMS: = Root mean square
